# The Origin and Development of Piji Pills: An Ancient Prescription of Traditional Chinese Medicine

**DOI:** 10.1155/2022/9090697

**Published:** 2022-09-12

**Authors:** Fudong Liu, Xiaochen Jiang, Chuanlong Zhang, Guibin Wang, Yi Li, Bo Pang

**Affiliations:** Guang'anmen Hospital, China Academy of Chinese Medical Sciences, Beijing 100053, China

## Abstract

**Objective:**

Ancient prescriptions of traditional Chinese medicine (TCM) are an important source for innovative drug research and development, which has garnered increasing attention in recent years. Piji Pills, an ancient TCM prescription, has a long history and remarkable clinical efficacy in the treatment of digestive disorders. Thus, the purpose of this study was to explore the origin and development of Piji Pills and to discuss the potential future direction of an ancient TCM prescription.

**Method:**

We analyzed the origin and development of the Piji Pills by reviewing literature records and their evolution in ancient books. We used a full-text database covering 2,090 TCM ancient books and implemented the full-text retrieval function based on Ulysses software. A full-text search was conducted using the keyword “Piji Pills” (“脾积丸” in Chinese). The results generated 128 pieces of literature from 35 ancient TCM books. In order to identify pertinent sections from the generated results, the results were proofread by two independent authors (Fudong Liu and Xiaochen Jiang) who had sufficient experience concerning ancient books. The developmental process of the Piji Pills was divided into early, late, and modern times. With the approach of statistical methods and chronological description, we manually searched, indexed, and transformed 2,090 ancient TCM books.

**Result:**

From the time Piji Pills were first proposed, the records in ancient books became increasingly detailed, providing an in-depth discussion of their composition, dosage, and action mechanisms. In modern times, the research on key drugs found in Piji Pills has made a great contribution to clinical practice. However, the compound research on Piji Pills is still relatively superficial and requires further in-depth study.

**Conclusions:**

In this study, statistical methods were used to chronologically clarify the developmental process of Piji Pills. We found that the Piji Pills were widely used and had a significant advantage in the treatment of digestive system diseases. In-depth knowledge mining of ancient books could potentially promote the theoretical innovation of TCM and the research of new drugs.

## 1. Introduction

The unique cognitive system and empirical methodology of traditional Chinese medicine (TCM) have evolved over millennia, and its theoretical system has consistently improved overtime. Ancient books are considered important wisdom carriers, depicting the achievements of TCM. They contain rich connotations that guide the continuous development and progress of TCM. One such example is the discovery of artemisinin by Tu Youyou, a Chinese pharmaceutical chemist and malariologist who was searching for a traditional malaria cure and was inspired by an ancient Chinese medical book (Zhou Hou Bei Ji Fang) and ultimately isolated and extracted artemisinin, an antimalaria drug. This was a breakthrough in twentieth-century medicine and later won the Nobel Prize [1]. Fan et al. confirmed that cepharanthine, an alkaloid from the TCM cephalothin, was a potential drug for treating 2019-nCoV infection [2]. Furthermore, a UK report addressed to the ministers on the statutory regulation of acupuncture practitioners, herbal medicine, TCM, and other traditional medicines indicated that the recorded history of traditional use should be assessed and incorporated into the existing evidence database to support the efficacy and safety of herbal/traditional medicines and acupuncture [3]. Directives by the European Parliament and the Council of the European Union on traditional herbal products have stated that long-term use of traditional medicinal products reduces the need for clinical trials, as the efficacy of medicinal products is plausible based on their long-standing use and experience [4]. Furthermore, the Law of the People's Republic of China on TCM has stated that it is essential to provide information on clinical safety studies when applying for drug approval numbers for Chinese medicine compound preparations that are derived from ancient classical prescriptions that meet the state requirements [5].

Due to dietary changes, the incidence of digestive system diseases has gradually increased and imposes an enormous economic burden on both the patient and the healthcare system. Chinese and Western medicine combinations have significant advantages in the treatment of digestive system diseases; for example, some experiments have confirmed the efficacy of Chinese medicine in the treatment of elicobacter pylori infection [6], ulcerative colitis [7], and intestinal flora disorders [8].

Piji Pills have remarkable clinical effects in the treatment of digestive system diseases. Zhai et al. confirmed the potential value of *β*-elemene, isolated from the Curcuma Rhizoma (Ezhu), for its anticancer effects [9]. Zhang et al. using network pharmacology and molecular docking technology, found that Piji Pills played a role in the treatment of pancreatic cancer through multicomponent, multtarget, and multichannel approaches [10].

We are conducting a randomized controlled clinical trial in metastatic pancreatic cancer patients, using prescription medication containing the key compositional compounds found in the Piji Pills, to further validate its efficacy and safety (trial registration number: ClinicalTrials.gov Identifier ChiCTR2000032875). It is, therefore, imperative to systematically review the classical medical literature to analyze the origins and development of the Piji Pills and to further explore the sources of innovation and the development of TCM.

## 2. Materials and Methods

### 2.1. Literature Search and Data Collection

The author independently constructed a full-text database covering 2,090 TCM ancient books and implemented the full-text retrieval function based on Ulysses software. A full-text search was conducted using the keyword “Piji Pills” (“脾积丸” in Chinese). In order to identify pertinent sections, the results were proofread by two independent authors (Fudong Liu and Xiaochen Jiang) who had sufficient experience concerning ancient books. Thereafter, we manually indexed and classified the pertinent written sections. For data format compatibility (CSV, ARFF), required by the data mining system, we used Microsoft Excel for Mac 16.62 (22061100) for data entry purposes. CSV data included eight entries from A to H: A dynasty, B author, C books, D original text, E drugs, F disease, G dose, and H dosage form. Reference WHO international standard terminologies on traditional Chinese medicine regulated TCM standard terminology [11].

### 2.2. Search Result

**Figure 1:**
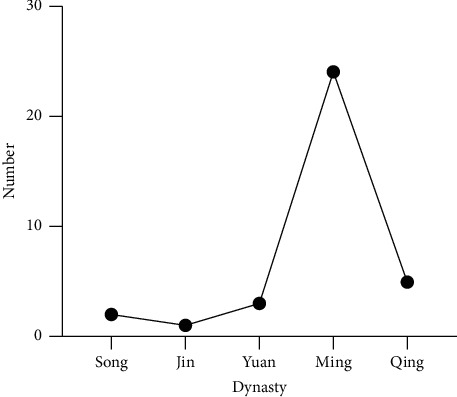
The number of ancient records of Piji Pills by dynasties from Song to Qing.

We reviewed more than 128 pieces of literature from 35 books (Figure 1), generated from our database of 2,090 ancient TCM books. Our area of focus was full literature describing the history of Piji Pills. We used the following statistical methods in our literature search: frequency statistics, complex network analysis, and association rules, to sort through the development, main symptoms, prescription principle, and Piji Pills' dosage. The origin, development, and future direction were discussed, taking into consideration the knowledge of TCM theory and recent research related to Piji Pills, which were divided into early, middle, and modern times. We aimed to provide new ideas and methods for the study of famous ancient TCM prescriptions.

## 3. Result and Discussion

### 3.1. Early Stage

The early Piji Pills records in ancient TCM books stretched from the Song dynasty (960–1279 AD) to the Yuan dynasty (1271–1368 AD), during which period the ancient Chinese medical texts not only achieved a break-through starting from scratch but also made great progress in the composition of drugs and the treatment scope of diseases (Table 1).

During the Song dynasty (960-1279 AD), the government attached great importance to the development of medicine. The first record of Piji Pills appeared in Sheng Ji Zong Lu (1117 AD), which was compiled by the government. The book contained nearly 20,000 prescriptions, including prescriptions from ancient medical texts, folk experiences, and prescriptions provided by medical practitioners. Piji Pills recorded in this book were composed of Old Rice (Chen Cang Mi), Citri Reticulatae Pericarpium Viride (Qing Pi), Crotonis Fructus (Ba Dou), Sparganii Rhizoma (San Leng, including Shi San Leng, Jizhua San Leng, and Jing San Leng), Curcumae Rhizoma (E Zhu), and Arecae Semen (Bing Lang). It was mainly used to treat the disease “Masses in the spleen” (Pi Qi), manifested by abdominal swelling, epigastric distension, jaundice, dry mouth, weight loss, reduced diet, chest tightness and intermittent fever. The disease, in terms of symptoms, resembled the digestive systems of malignant tumors and was treated by Piji Pills. However, the absence of a more detailed disease description in the book limited its further application. The Piji Pills were later described in Renzhai Zhizhi Fanglun, 1264 AD, where it was recorded that Piji Pills could potentially be used for the treatment of malaria, dyspepsia, indigestion, and constipation, among others. The prescription composition of Piji Pills had been “updated.” Old Rice (Chen Cang Mi) and Arecae Semen (Bing Lang) were removed; Alpiniae Officinarum rhizoma (Gao Liang Jiang), Gleditsiae Sinensis Fructus (Da Zao Jiao), and Plant Soot (Bai Cao Shuang) were added. Thereafter, most of the ancient books described Piji pills based on this prescription composition.

During the Jin and Yuan dynasties, TCM developed rapidly. Four famous Jin and Yuan healers (Zhang Congzheng, Zhu Zhenheng, Liu Wansu, and Li Gao) made a great impact on the development of TCM. During this period, descriptions of Piji Pills were gradually enriched. Four ancient books recorded the composition, dosage, and main symptoms of this prescription. Convenient prescriptions for doctor's cowardice (Yi Fang BianNuo), written by Li Gao, the founder of the “Spleen-Stomach Theory,” further expanded the therapeutic effect of Piji Pills. The book recorded that Piji pills were used to treat indigestion, acid reflux, vomiting, pediatric malnutrition, and abdominal pain in women caused by Qi-blood disharmony, covering internal medicine, pediatrics, and gynecological diseases. Li Gao added a Qi-regulating herb, CaryophylliFlos (Ding Xiang), to the pills, reflecting the academic view that “the spleen and stomach are the pivots of Qi movement.” Influenced by Li Gao, this drug was later widely used by doctors.

### 3.2. Late Stage

The development of Chinese medical theory further matured from the Ming dynasty to the Qing dynasty (1368 AD-1644 AD). Especially toward the end of the Qing dynasty, the idea of combining Chinese and Western medicine began to seep into the minds of doctors as Western medicine was introduced into China. During this time, the development and application of Piji Pills became more widespread. Many monographs on pediatrics, internal medicine, and miscellaneous diseases elaborate on the indications for diseases and the medicine dosage.

**Figure 2:**
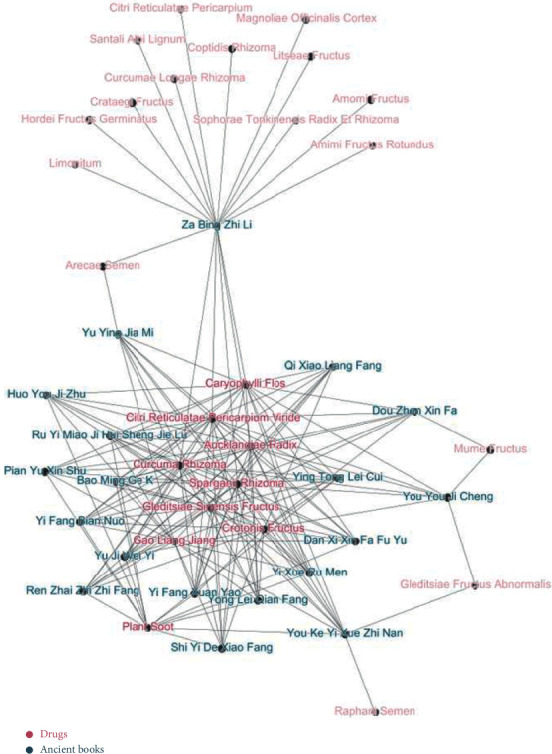
The complex network of core drug composition of Piji Pills and related ancient books.

Twenty-two pieces of literature recorded the drug composition of Piji Pills. Based on the analysis of this literature using the Gephi complex network (Figure 2), it was found that the core components of Piji Pills were eight herbs: Aucklandiae Radix (Mu Xiang), Crotonis Fructus (Ba Dou), Alpiniae Officinarum Rhizoma (Gao Liang Jiang), Sparganii Rhizoma (San Leng), Curcumae Rhizoma (E Zhu), Citri Reticulatae Pericarpium Viride (Qing Pi), Gleditsiae Sinensis Fructus (Da Zao Jiao), and Plant Soot (Bai Cao Shuang). Only a small number of books differ slightly in composition, including Pediatric parenting methods (YuYing JiaMi), cases of miscellaneous diseases (ZaBing ZhiLi), complete book on paediatrics (YouYou JiCheng), experience in treating pox and rash (DouZhen XinFa) and guidebook of pediatrics (YouKe YiXueZhiNan).

**Figure 3:**
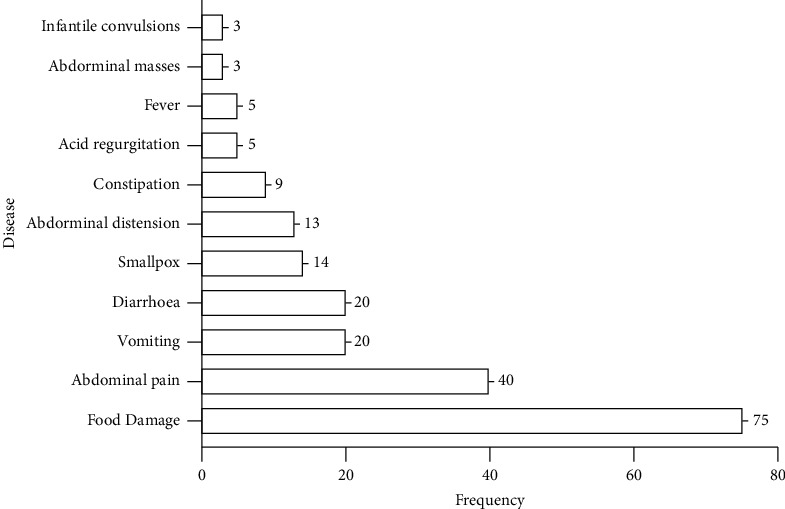
Frequency analysis of the main diseases (symptoms) treated with Piji Pills.

To fully discover the main diseases treated by Piji Pills and to more accurately guide clinical application, we cleaned, transformed, and summarized the data on the diseases (symptoms) recorded in the literature. We then used VBA programs to disaggregate the symptoms and perform descriptive statistics (Figure 3), which showed that most of the symptoms were closely related to the digestive system.

Wan Mizhai, a Yuan dynasty physician who was renowned in pediatrics and gynecology, often used the Piji Pills to treat pediatric pox and rash, dyspepsia, indigestion, and infantile convulsion. In You Ke Fa Hui, it was written that “the stomach is known as ‘the sea of water and food', and the intestine is ‘the way of water and food'. Diarrhea is a disease caused by damage to the stomach and intestines. If there is no pathogenic qi in the stomach and intestines, food and water will not become bad, and the person will have no disease. If food retention stays in the stomach and intestines, diarrhea will occur, just like more water in a container will flow out. If diarrhea is to be treated, the source of the disease must be found. The spleen jack pill is the medicine that can get rid of food retention at its source.” This is one of the few discussions in ancient books of TCM on the mechanism of Piji Pills in treating digestive disorders, and it emphasizes the mechanism of Piji Pills with the spleen and stomach as its core. The famous pediatrician Zhou Zhen of the Qing dynasty was also inspired by this and used the Piji Pills to treat diarrhea, abdominal pain, night cries, and other pediatric diseases caused by food accumulation.

According to basic TCM theory, the spleen and stomach are the pivots of Qi movement. If Qi movement in the spleen and stomach is not properly regulated, abdominal distension and pain will occur. If there is an imbalance in the ascending and descending of Qi movement, Qi counterflow will lead to nausea, vomiting, acid reflux, and constipation. If the spleen-yang is trapped, diarrhea will follow. If Qi is blocked for a prolonged period of time, it will further develop into tangible accumulation. Therefore, with the use of a large number of herbs that promote the movement of Qi, the Piji Pills have become increasingly effective in the treatment of digestive disorders, especially after Li Gao added Caryophylli Flos (Ding Xiang) to the composition of the medicine.

Based on more complete records of Piji Pills in the Ming and Qing dynasties, combined with those from the Song, Jin, and Yuan dynasties, the dosages of Piji Pills' herbs were counted (Table 2). The analysis covered the maximum, minimum, and average amounts of each herb, the occurrence frequency of the maximum and minimum amounts, the highest occurrence frequency of the same dosage, and the specific dosage in ancient books. In our research, we found that over the last millennia since its development, there were significant differences in the drug's weights and measurements. To provide a standard for the modern clinical application of Piji Pills, we converted the units recorded in previous dynasties into “grams” (g). According to data provided in the History of Chinese Science and Technology—Weights and Measures Volume, one tael in the Song and Yuan dynasties was approximately 41.3 g in modern times, and one tael in the Ming and Qing dynasties was approximately 37.3 g in modern times.

In addition, there were some rare units of measure recorded in ancient books, making data conversion difficult. For example, the most frequent record of the amount of Gleditsiae Sinensis Fructus (Da Zao Jiao) was “3 ting,” which was recorded in seven books. We also searched the relevant books and found that Pro. Shi Shenghan, in his commentary on QiMin Yao Shu, believed that the unit of “ting” was equivalent to the modern measurement of “ingot” (one piece). So “3 ingots” were three pieces. At the same time, the dosage of the large soapwort was recorded as “3 qian” (11.19 g) in Dou Zhen Xin Fa and You You Ji Cheng. In summary, we assumed that the dosage of Gleditsiae Sinensis Fructus (Da Zao Jiao) was indicative of approximately15 g. The dosage of Plant Soot (Bai Cao Shuang) was calculated in a similar manner as previously mentioned. Eight books contained a dosage of “3 spoons,” and combined with the dosage of “3 qian” (11.19 g) in four books in the Ming and Qing dynasties, the dosage of Plant Soot (Bai Cao Shuang) was considered to be 12 g.

In terms of the maximum amount for each recipe, Sparganii Rhizoma (San Leng) and Curcumae Rhizoma (E Zhu) were the most commonly used. The minimum dosage of each herb was taken from You Ke Yi Xue Zhi Nan, written by Zhou Zhen in the Qing dynasty, which lacked statistical significance. In terms of average dosage, the dosage of Curcumae Rhizoma (E Zhu) was the largest, followed by Sparganii Rhizoma (San Leng), Citri Reticulatae Pericarpium Viride (Qing Pi), Alpiniae Officinarum Rhizom (Gao Liang Jiang), Crotonis Fructus (Ba Dou) and Aucklandiae Radix (Mu Xiang).

In regards to the dosage form, we screened 128 pieces of literature documenting Piji Pills and found that the dosage form used in 22 documents were all pills. We also searched for information on pills in ancient texts. The most representative one was Li Gao's comment in his Yong Yao Fa Xiang that “pills are slow, soothing, and healing.” Modern pharmacological studies have also confirmed the ability of solid oral agents to deliver live phages to the gastrointestinal tract [12], and the release of active ingredients from the pills was relatively slow in the intestinal fluid environment simulated by the bicarbonate buffered dissolution medium [13]. Therefore, the application of pills not only allowed the active ingredients to remain in the body for a longer period of time but also allowed for more precise targeting of herbal medicine. In the 2015 edition of the Chinese Pharmacopoeia, approximately 25.71% of the dosage forms were pills, indicating the wide usage of pills in TCM clinics.

### 3.3. Modern times

Modern times refer to the time period from the beginning of the Republic of China (1912 AD-1949 AD) to the present day. In the early stage of this period, TCM was challenged by western medicine. At the beginning of the twentieth century, the increasing attention to western medicine hindered the development of TCM [14]. Since 1949, under the guidance of scientific theory and technology, TCM has been able to gradually carry out in-depth research and perform experimental studies [15]. The study of various drugs found in Piji pills had also entered a new stage from macro to micro. For example, Chen et al. confirmed that Curcumae Rhizoma (E Zhu) had great potential for anti-inflammatory, anticancer, hepatoprotective, and immunomodulatory purposes [16]. In China, *β*-elemene isolated from Curcumae Rhizoma (E Zhu), had been developed into a drug for the treatment of solid tumors and malignant effusion by intravenous injection, intraperitoneal or peritoneal perfusion. The drug is currently being tested in clinical trials in the United States [17]. Simultaneously, Chang et al. had demonstrated that the new compounds resulting from the combination of Curcumae Rhizoma (E Zhu) and Sparganii Rhizoma (San Leng) had better antitumor activity than the single herbs [18]. Therefore, it was generally believed that strengthening the research of TCM compound prescriptions would be conducive to and promote the process of new drug research and development. However, at present, the modern scientific connotation of the compound theory for Piji Pills has not yet been elucidated, due to the complexity of the active ingredients and the mere fact that their safety and efficacy need to be further verified. Therefore, modern research on the Piji Pills is still at a blank stage.

### 3.4. Future Perspectives

Machine learning (ML) and artificial intelligence (AI) are developing rapidly in the medical field, which can be beneficial to both medical staff and patients. The IBM Corporation has developed an AI-assisted decision-making system, known as Watson for Oncology (WFO), that is based on cognitive computing and includes medical literature, patents, genomics information, and chemical and pharmacological data. Several studies have confirmed that WFO may be a helpful tool for cancer treatment decision-making [19–21]. Moreover, in the field of TCM, Fang et al. built a high-throughput experiment and reference-guided TCM database, named HERB, that could strongly support the modernization of TCM [22]. Furthermore, Xu et al. developed an Encyclopedia of TCM (ETCM), which contains comprehensive and standardized information on the commonly used herbs and formulas of TCM as well as their ingredients [23]. Huang et al. constructed the TCM Integrated Database (TCMID), which records TCM-related information collected from different resources and through a text-mining method [24, 25]. These databases are highly recognized by TCM researchers and would make a significant contribution to the modernization of TCM research.

However, most of these studies were conducted based on modern research and literature. The TCM classics and literature cannot be taken as the object of these studies because the ancient TCM books have accumulated a lot of data over the last millennia since its initial development. A lot of research achievements in prescriptions, herbs, ingredients, and other TCM-related information were dispersedly recorded in books and journals, which hindered the systematic research and application of TCM.

In 2018, the State Administration of TCM (SATCM) selected 100 ancient classical prescription records from more than 100,000 prescriptions recorded in 103 medical books. Currently, several documentary studies on classically famous prescriptions are being conducted. TCM has been garnering increasing attention, which has increased the need for TCM-related data resources. Our study had the following strengths. First, our database was extensive; we retrieved the information from a large number of ancient TCM books, including ancient books from the Song, Jin, Yuan, Ming, and Qing dynasties, which eliminated the deviation caused by historical factors and provided greater literature comprehension. Second, we designed an indexing manual and a manual classification strategy for ancient discourse and introduced statistical methods to provide a more objective picture of what was written in ancient texts about Piji Pills, eliminating the bias caused by limited researcher understanding.

Despite the aforementioned strengths, our study had some limitations. First, only 128 documents were included in the final review and analysis, and the maneuverability of the indexing manual and classification strategy designed in this study required more testing data. Second, this study used statistical methods to explore the mining of ancient TCM books, which needed to be further combined with ML and AI techniques. Third, this study has only clarified the information on the composition, dosage, and symptoms of Piji Pills; future research on the mechanism of Piji Pills needs to be conducted to promote the modernization of Chinese medicine.

## 4. Conclusions

Piji Pills, an ancient TCM prescription, has a long history and was inherited and developed in the Ming and Qing dynasties. This study applied statistical methods to extensively investigate and review ancient TCM books, using a chronological approach to sort through the results of the analysis. Based on the study results, we found that the core components of Piji Pills were “Aucklandiae Radix (Mu Xiang), Crotonis Fructus (Ba Dou), Alpiniae Officinarum Rhizoma (Gao Liang Jiang), Sparganii Rhizoma (San Leng), Curcumae Rhizoma (E Zhu), CitriReticulatae PericarpiumViride (Qing Pi), Gleditsiae Sinensis Fructus (Da Zao Jiao), and lPant Soot (Bai Cao Shuang)”, with Sparganii Rhizoma (San Leng) and Curcumae Rhizoma (E Zhu) being the key drugs. Furthermore, we found it was used in clinical practice in the form of pills and had significant advantages in the treatment of digestive disorders. Modern scientific research supports the views expressed in ancient texts. This study concluded that promoting the deep integration of information technology with the content mining of ancient TCM books could facilitate theoretical innovation and the creation of new medicines.

## Figures and Tables

**Table 1:** Summary of the main contributions to the records of Piji Pills in the early stage.

Representative books	Author	Time	Contributions
Sheng Ji Zong Lu	Zhao ji	1117 AD	The first record of “Piji Pills,” the main symptoms, composition, dosage, and production method.
Ren ZhaiZhiZhi Fang Lun	Yang Shi-ying	1264 AD	The meaning of “Piji” was expanded and the scope of treatment was enriched. The composition gradually matured and stabilized.
YiFang BianNuo	Li Gao	1266 AD	Based on the Treatise on spleen and stomach, Caryophylli Flos was added to the composition of Piji Pills. This prescription composition was widely used by doctors of later generations.

**Table 2:** Dosage and frequency of occurrence of Piji Pills.

Dugs	Minimum dosage and frequency		Maximum dosage and frequency		Average/g	Most common doses and frequencies	
	Minimum dosage/g	Frequency/no.	Maximum dosage/g	Frequency/no.		Dosage/g	Frequency/no.
Sparganii Rhizoma	1.87	1	82.6	1	45.59	74.6	9
Curcumae Rhizoma	1.87	1	123.9	1	58.21	111.9	6
Citri Reticulatae Pericarpium Viride	1.87	1	41.3	1	27.28	37.3	12
Aucklandiae Radix	2.98	1	20.65	1	15.60	18.65	14
Alpiniae Officinarum Rhizoma	1.87	1	55.95	1	18.46	18.65	14
Crotonis Fructus	1.87	1	20.65	1	16.94	18.65	10

## Data Availability

All data were included in the manuscript and there was no restriction on availability.
